# Pre-therapeutic microglia activation and sex determine therapy effects of chronic immunomodulation

**DOI:** 10.7150/thno.64022

**Published:** 2021-08-19

**Authors:** Gloria Biechele, Tanja Blume, Maximilian Deussing, Benedikt Zott, Yuan Shi, Xianyuan Xiang, Nicolai Franzmeier, Gernot Kleinberger, Finn Peters, Katharina Ochs, Carola Focke, Christian Sacher, Karin Wind, Claudio Schmidt, Simon Lindner, Franz-Josef Gildehaus, Florian Eckenweber, Leonie Beyer, Barbara von Ungern-Sternberg, Peter Bartenstein, Karlheinz Baumann, Mario M. Dorostkar, Axel Rominger, Paul Cumming, Michael Willem, Helmuth Adelsberger, Jochen Herms, Matthias Brendel

**Affiliations:** 1 Dept. of Nuclear Medicine, University Hospital of Munich, LMU Munich, Munich, Germany.; 2 DZNE - German Center for Neurodegenerative Diseases, Munich, Germany.; 3 Institute of Neuroscience, Technical University of Munich, Munich, Germany.; 4 Department of Diagnostic and Interventional Neuroradiology, Klinikum rechts der Isar, Technical University of Munich, Munich, Germany.; 5 Metabolic Biochemistry, Biomedical Center (BMC), Faculty of Medicine, Ludwig-Maximilians-Universität München, Munich, Germany.; 6 Institute for Stroke and Dementia Research, University Hospital of Munich, LMU Munich, Munich, Germany.; 7 ISAR Bioscience GmbH, 82152 Planegg, Germany.; 8 SyNergy, University of Munich, Munich, Germany.; 9 Roche Pharma Research and Early Development, Neuroscience Discovery, Roche, Innovation Center Basel, F. Hoffmann-La Roche Ltd., Basel, Switzerland.; 10 Center for Neuropathology and Prion Research, Ludwig-Maximilians-University of Munich, Munich, Germany.; 11 Dept. of Nuclear Medicine, Inselspital Bern, Bern, Switzerland.; 12 School of Psychology and Counselling, Queensland University of Technology, Brisbane, Australia.

**Keywords:** pioglitazone, TSPO-PET, *App^NL-G-F^* mice, PS2APP mice, microglia, sex, prediction

## Abstract

Modulation of the innate immune system is emerging as a promising therapeutic strategy against Alzheimer's disease (AD). However, determinants of a beneficial therapeutic effect are ill-understood. Thus, we investigated the potential of 18 kDa translocator protein positron-emission-tomography (TSPO-PET) for assessment of microglial activation in mouse brain before and during chronic immunomodulation.

**Methods:** Serial TSPO-PET was performed during five months of chronic microglia modulation by stimulation of the peroxisome proliferator-activated receptor (PPAR)-γ with pioglitazone in two different mouse models of AD (PS2APP, *App^NL-G-F^*). Using mixed statistical models on longitudinal TSPO-PET data, we tested for effects of therapy and sex on treatment response. We tested correlations of baseline with longitudinal measures of TSPO-PET, and correlations between PET results with spatial learning performance and β-amyloid accumulation of individual mice. Immunohistochemistry was used to determine the molecular source of the TSPO-PET signal.

**Results:** Pioglitazone-treated female PS2APP and *App^NL-G-F^* mice showed attenuation of the longitudinal increases in TSPO-PET signal when compared to vehicle controls, whereas treated male *App^NL-G-F^* mice showed the opposite effect. Baseline TSPO-PET strongly predicted changes in microglial activation in treated mice (R = -0.874, p < 0.0001) but not in vehicle controls (R = -0.356, p = 0.081). Reduced TSPO-PET signal upon pharmacological treatment was associated with better spatial learning despite higher fibrillar β-amyloid accumulation. Immunohistochemistry confirmed activated microglia to be the source of the TSPO-PET signal (R = 0.952, p < 0.0001).

**Conclusion:** TSPO-PET represents a sensitive biomarker for monitoring of immunomodulation and closely reflects activated microglia. Sex and pre-therapeutic assessment of baseline microglial activation predict individual immunomodulation effects and may serve for responder stratification.

## Introduction

Neuroinflammation is now recognized as an inherent part of the Alzheimer's disease (AD) pathology 1. The key players of neuroinflammation in AD are activated microglia and astrocytes 2. Although it is still unclear if beneficial or detrimental effects of neuroinflammation dominate in the (patho)physiology of AD, there is considerable interest in integrating the modulation of neuroinflammation into novel treatment strategies against AD 3. Preclinical studies showed that immunomodulation by peroxisome proliferator-activated receptor (PPAR)-γ using the antidiabetic compound pioglitazone rescues neuronal spine density 4 and spatial learning performance 5 in mouse AD models. However, a large human trial with pioglitazone in mild cognitive impairment due to AD was terminated after an interim analysis showing lack of efficacy 6. Hence, the discrepancies between beneficial effects in preclinical studies and lacking efficacy in humans deserve detailed inquiry, with the objective of uncovering the salient factors accounting for the failure of PPARγ stimulation in clinical translation.

TSPO-PET is increasingly used to monitor therapy-related changes of microglial activation in humans 7 and rodent models 8. In this regard, the TSPO ligand ^18^F-GE-180 is proven effective for robust imaging of microglial activation in a mouse model of amyloidosis, and shows the normalization of TSPO binding upon treatment with a neurotrophin receptor ligand that ameliorates hyperphosphorylation and misfolding of tau, and rescues the consequent neurite degeneration 9. Our previous data revealed excellent agreement between ^18^F-GE-180 PET quantitation and immunohistochemistry of microglial markers 10, 11, thus indicating its potential to access and predict PPARγ stimulation effects *in vivo*.

Therefore, we aimed in this study to test the hypothesis that TSPO-PET with ^18^F-GE-180 is a suitable tool for monitoring anti-neuroinflammatory responses to chronic immunomodulation in AD mouse models. We furthermore tested the hypothesis that microglial activation by TSPO-PET predicts therapy-related changes and outcome parameters. Furthermore, we tested for effects of mouse sex on immunomodulation. Finally, we used immunohistochemistry to validate *in vivo* PET findings and to confirm the cellular source of TSPO-PET signal alterations.

## Material and Methods

### Study design

All experiments were performed in compliance with the National Guidelines for Animal Protection, Germany, with approval of the local animal care committee of the Government of Oberbayern (Regierung Oberbayern) and overseen by a veterinarian. The experiments also complied with the ARRIVE guidelines and were carried out in accordance with the U.K. Animals (Scientific Procedures) Act, 1986 and associated guidelines, EU Directive 2010/63/EU for animal experiments. The chronic treatment study was performed in two different Aβ mouse models and a longitudinal PET imaging design was applied in both cohorts. Female PS2APP and wild-type mice had their baseline assessment at eight months of age and had follow-up PET imaging at 9.5, 11.5 and 13 months of age. Female and male *App^NL-G-F^* mice had their baseline assessment at five months of age and received follow-up PET imaging at 7.5 and 10 months of age. Cage randomization to pioglitazone treatment or control chow (vehicle) groups was initiated after the baseline PET scans, and treatments continued until after the terminal behavioural assessments. After recovering from the final PET scan, mice were transferred to the behavioural facility and rested for one week before initiation of Morris water maze (WM) testing of spatial learning. One week after the behavioural tests, mice were deeply anaesthetized prior to transcardial perfusion and fixation with 4% paraformaldehyde. We then harvested and processed the brains for immunohistochemical and biochemical analyses (randomized hemispheres). Group comparisons of longitudinal Aβ-PET monitoring and detailed Aβ analyses by immunohistochemistry and biochemistry of the same cohort are reported in a separate manuscript 12. Shared data points between both manuscripts are indicated and cited.

### Animal Models and Statistical Power Analysis

The transgenic B6.PS2APP (line B6.152H) is homozygous for human presenilin (PS) 2, the N141I mutation, and the human amyloid precursor protein (APP) K670N/M671L mutations 13. Homozygous B6.PS2APP mice show first appearance of plaques in the cerebral cortex and hippocampus at 5-6 months of age 14. The knock-in mouse model *App^NL-G-F^* carries a mutant APP gene encoding the humanized Aβ sequence (G601R, F606Y, and R609H) with three pathogenic mutations, namely Swedish (KM595/596NL), Beyreuther/Iberian (I641F), and Arctic (E618G). Homozygotic *App^NL-G-F^* mice progressively exhibit widespread Aβ accumulation from two months of age 15, 16. Both transgenic models were generated on a C57Bl/6 background, which also served for wild-type controls.

Required sample sizes were calculated by G*power (V3.1.9.2, Kiel, Germany), based on assumptions for a type I error α=0.05 and a power of 0.8 for group comparisons. A drop-out rate of 10% per time-point was assumed and a treatment effect causing 5% change in the PET signal was considered significant. Estimations were based on PET measures in previous investigations with the same mouse models 10, 17. Calculated sample sizes at baseline were n = 14 for PS2APP, n = 8 for wild-type, and n = 9 per sex for *App^NL-G-F^*.

### PET Imaging

For all PET procedures, radiochemistry, data acquisition, and image pre-processing were conducted according to an established, standardized protocol 18. In brief, ^18^F-GE-180 TSPO-PET recordings (average dose: 11.5 ± 2.2 MBq) with an emission window of 60-90 min after injection were performed for assessment of cerebral TSPO expression. Aβ-PET recordings (^18^F-florbetaben average dose: 12.2 ± 2.0 MBq) with an emission window of 30-60 min after injection were obtained to measure fibrillar cerebral amyloidosis, as reported elsewhere 12. Isoflurane anesthesia was induced before tracer injection and maintained to the end of the imaging time window. Mice with different genotype and treatment arm were examined simultaneously, with random placement in a four-mouse imaging chamber, thus with exposure to an equal level of isoflurane during the PET recording. All image analyses were performed using PMOD (version 3.5; PMOD technologies, Basel, Switzerland). Static 30-60 min (Aβ-PET) and 60-90 min (TSPO-PET) datasets were co-registered to tracer specific templates (genotype specific) by a manual rigid-body transformation (TX_rigid_) 18. In the second step, a reader-independent affine co-registration to the tracer-specific template was performed 18. Here, the initial manually fused images were further normalized by non-linear brain normalization (TX_BN_) via the PMOD brain normalization tool (equal modality; smoothing by 0.6 mm; nonlinear warping; 16 iterations; frequency cutoff 3; regularization 1.0; no thresholding). The concatenation of TX_rigid_ and TX_BN_ was then used to obtain optimal resampling with a minimum of interpolation. Normalization of injected radioactivity was performed by the previously validated myocardium correction method 19 for TSPO-PET and by previously established white matter 18 (PS2APP) and periaqueductal grey 17 (*App^NL-G-F^*) reference regions for Aβ-PET. Thus, the primary endpoints of PET consisted of myocardium-adjusted standardized uptake values (SUV_H_) for TSPO-PET and intracerebral reference-based standardized uptake value ratios (SUVr) for Aβ-PET. TSPO- and Aβ-PET estimates (per time-point and rate of change) deriving from the same neocortical target VOI (15 mm³) were extracted and compared between treatment and vehicle groups as well as between transgenic mice and wild-type controls by mixed linear models. The TSPO-PET z-score of each individual transgenic mouse at each time-point was calculated by subtraction of the mean TSPO-PET value of vehicle treated, age-matched wild-type mice and division by the standard deviation of wild-type mice (z-score = [mean_TG_ - mean_WT-Veh_]/SD_WT-Veh_). The z-score deviation per time was defined as a TSPO-PET AUC 20 and served as an index for microglial activation during the observation time period. For the association analysis between baseline TSPO-PET and changes of TSPO-PET over time (Δ z-score = rate of change), we additionally extracted VOIs from the Mirrione atlas 21 to allow evaluation of multiple brain regions. The large cortex VOI of the atlas was divided into motor/sensory, auditory/visual and entorhinal/piriform cortices to allow evaluation within functionally similar compartments. We applied a false discovery rate correction for multiple comparisons.

### Water maze

Two slightly different Morris water maze tasks were applied due to facility changes between the investigation of PS2APP and *App^NL-G-F^* cohorts. We used a principal component analysis of the standard read-outs of each water maze task to generate a robust read-out for correlation analyses 22. Thus, one quantitative index of water maze performance per mouse was calculated via dimension reduction and correlated with PET imaging. The experimenter was blind according to the phenotype of the animals. Water maze results were also used as an endpoint in the dedicated manuscript on Aβ-PET in both mouse models 12.

PS2APP and age-matched wild-type mice were subjected to a modified water maze task as described previously 20, 23-25 yielding escape latency, distance to the correct platform, and correct choice of the platform as read outs. Mice had to distinguish between two visible platforms, one of which was weighted in such a manner that it would float when the mouse climbed on (correct choice), while the other would sink (wrong choice). The correct platform was always located at the same spot in the maze, while the wrong platform as well as the site from which the mice were released into the maze were varied in a pseudorandom fashion. Visual cues on the walls of the laboratory provided orientation. Trials were terminated if the mouse had failed to reach one of the platforms within 30 sec (error of omission). In this case, or in case of a wrong choice, the experimenter placed the mouse on the correct platform. After a three-day handling period, water maze training was performed on five consecutive days, with five trials per day, which were conducted 2-4 minutes apart. Memory performance was assessed by measuring the escape latency at each day of training and by the travelled distance at the last training day. For measuring escape latency, we calculated the summed average time of all trials from the start point to attaining one of the platforms. On the sixth day, the correct platform was placed in the opposite quadrant of the maze to confirm that the mice indeed used spatial cues rather than rule-based learning to find it. Trials were filmed with a video camera and the swimming trace was extracted using custom written LabView software (National Instruments).

*App^NL-G-F^* mice and 14 age- and sex-matched wild-type mice underwent a common Morris water maze test, which was performed according to a standard protocol with small adjustments 26 as previously described 17. In brief, the first day was used for acclimatization with a visible platform (five minutes per mouse). The mice then underwent five training days where each mouse had to perform four trials per day with the platform visible at the first training day and the platform hidden under water for all other training days. The test day was set by only one trial with complete removal of the platform. The maximum trial length on all training and test days was set to a maximum of 70 seconds. The video tracking software EthoVision^®^ XT (Noldus) was used for analyses of escape latency, the platform frequency and attendance in the platform quadrant at the probe trial.

The principal component of the water maze test scores was extracted from three spatial learning readouts (PS2APP: escape latency, distance, platform choice; *App^NL-G-F^*: escape latency, frequency to platform, time spent in platform quadrant) using SPSS 26 statistics (IBM Deutschland GmbH, Ehningen, Germany). Prior to the PCA, the linearity of the relationship of the data was tested by a correlation matrix, and items with a correlation coefficient < 0.3 were discarded. The Kaiser-Meyer-Olkin (KMO) measure and Bartlett's test of sphericity were used to test for sampling adequacy and suitability for data reduction. Components with an Eigenvalue > 1.0 were extracted and a varimax rotation was selected.

### Immunohistochemistry

Iba-1 and CD68 immunohistochemistry was performed as described previously 17, 27 and the group comparisons between treatment and vehicle groups are reported in the accompanying manuscript 12. Correlation analyses were performed between TSPO-PET and Iba-1/CD68 quantitation. Groups of n = 4-5 PS2APP and *App^NL-G-F^* mice per treatment and vehicle groups with a successful TSPO-PET scan prior to immunohistochemistry were subjected to this analysis. In brief, we performed a standard free-floating immunofluorescence protocol with cortex areas matching the PET brain regions of interest. As previously described, perfusion-fixed 50-µm thick brain sections were rinsed either overnight or for 48 h in PBS with 0.2% Triton X-100 containing one of the following primary antibodies: rabbit monoclonal Iba-1 (1:500. Wako: 19-19741), or rat monoclonal CD68 (1:500. Bio-rad: MCA1857). After washing in PBS, sections were then incubated in a combination of three secondary antibodies (Alexa 488 goat anti-rabbit, Alexa 594 goat anti-mouse). A detailed analysis of Aβ-plaques (methoxy-X04 and NAB223) of this cohort is reported in the accompanying manuscript 12.

### Statistics

Group differences (i.e. between treatment groups or sexes) in TSPO-PET trajectories over time were determined using linear mixed models using the lmer package in the R statistical software, including a random intercept. Note that we selected models including either linear or quadratic time effects based on best model fit (i.e. lower Akaike Information Criterion for better model fit).

Association analyses were performed between PET, water maze, and immunohistochemistry scores. Pearson's coefficient of correlation (R) was calculated after confirming normal distribution by a Kolmogorov-Smirnow test. Correlation analysis was performed between TSPO-PET baseline (z-score) and the rate of change of TSPO-PET signal (Δ z-score). This analysis was performed in the cortical target region and in a separate analysis of the full Mirrione atlas set of VOIs 21. False discovery rate correction was applied for the multi-region analysis. The rate of change of TSPO-PET (Δ z-score) was correlated with the principal component of the water maze task to investigate potential associations of the PPARγ stimulation treatment effect with spatial learning performance. The index of microglial activity during a certain time-period (AUC) was correlated with the Aβ-PET rate of change to test the hypothesis of Aβ removal by activated microglia 28. Immunohistochemistry quantification (Iba-1 and CD68) in the cortex was correlated with the cortical TSPO-PET signal of the terminal time-point.

## Results

### TSPO-PET detects altered microglia activation during chronic PPARγ stimulation

**Figure 1:**
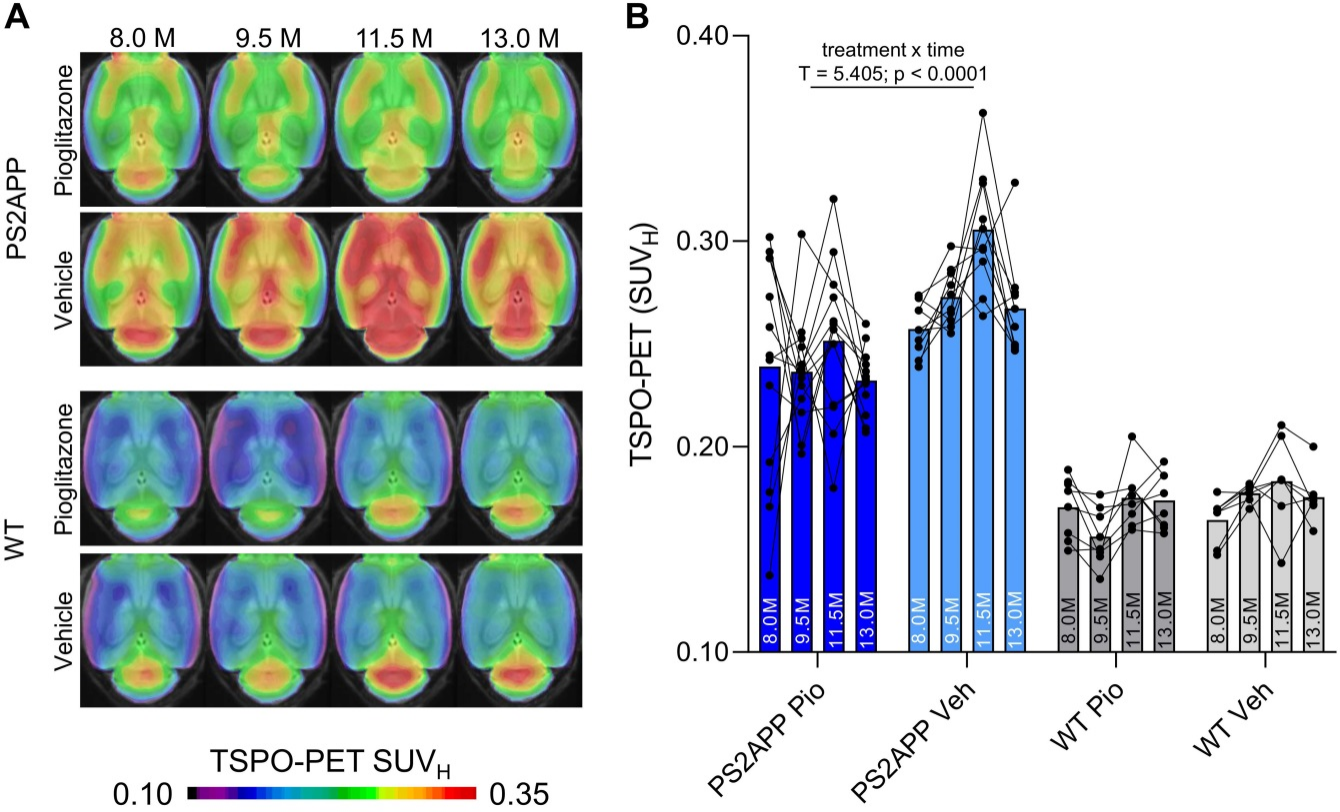
TSPO-PET monitoring of chronic pioglitazone treatment in PS2APP and wild-type (WT) mice. (**A**) Axial images show group levels of the ^18^F-GE-180 TSPO-PET signal (myocardium scaled standardized uptake value, SUV_H_) at different ages in treatment and vehicle groups, projected upon a standard MRI anatomic template. Baseline scans were performed prior to treatment initiation. (**B**) Individual time courses of the cortical TSPO-PET signal during the treatment period. Pio = pioglitazone treatment, Veh = vehicle treatment. Statistics derive from a linear mixed model. PS2APP pioglitazone n = 13, PS2APP vehicle n = 10, WT pioglitazone n = 8, WT vehicle n = 7.

First, we investigated whether effects of chronic PPARγ stimulation can be detected by TSPO PET in PS2APP mice and wild-type controls. Vehicle-treated PS2APP mice showed a strong increase over time of the TSPO-PET signal when compared to vehicle-treated wild-type mice between eight and 13 months of age, with a peak at 11.5 months (+52-67%, all time-points: p < 0.0001, **Figure 1**). The pre-therapeutic baseline TSPO-PET signal did not significantly differ between PS2APP mice with and without pioglitazone treatment (SUV_H_: 0.24±0.05 vs. 0.26±0.01, p = 0.647). However, PS2APP mice with pioglitazone treatment had an attenuated TSPO-PET signal at 9.5 (-13%, p = 0.0027), 11.5 (-17%, p = 0.0046), and 13.0 (-13%, p = 0.0071) months of age when compared to the increasing signal of age-matched vehicle-treated PS2APP mice (**Figure 1**). Linear mixed models revealed a main effect of treatment group on TSPO-PET across time-points (b/SE = -0.036/0.006, T = 5.405, p < 0.0001), controlling for age (i.e. quadratic effect) and random intercept. Individual PS2APP mice indicated a heterogeneous pharmacotherapy-related change in the TSPO-PET signal, which was already conspicuous during the first six weeks of treatment (range of change: -35 to +86%). Pioglitazone-treated wild-type mice manifested a slight decrease of the TSPO-PET signal after six weeks of treatment when compared to vehicle-treated wild-type mice (-12%, p = 0.013), and no such differences at the other time points.

### Chronic PPARγ stimulation changes microglial activation independent of APP overexpression but dependent on sex

**Figure 2:**
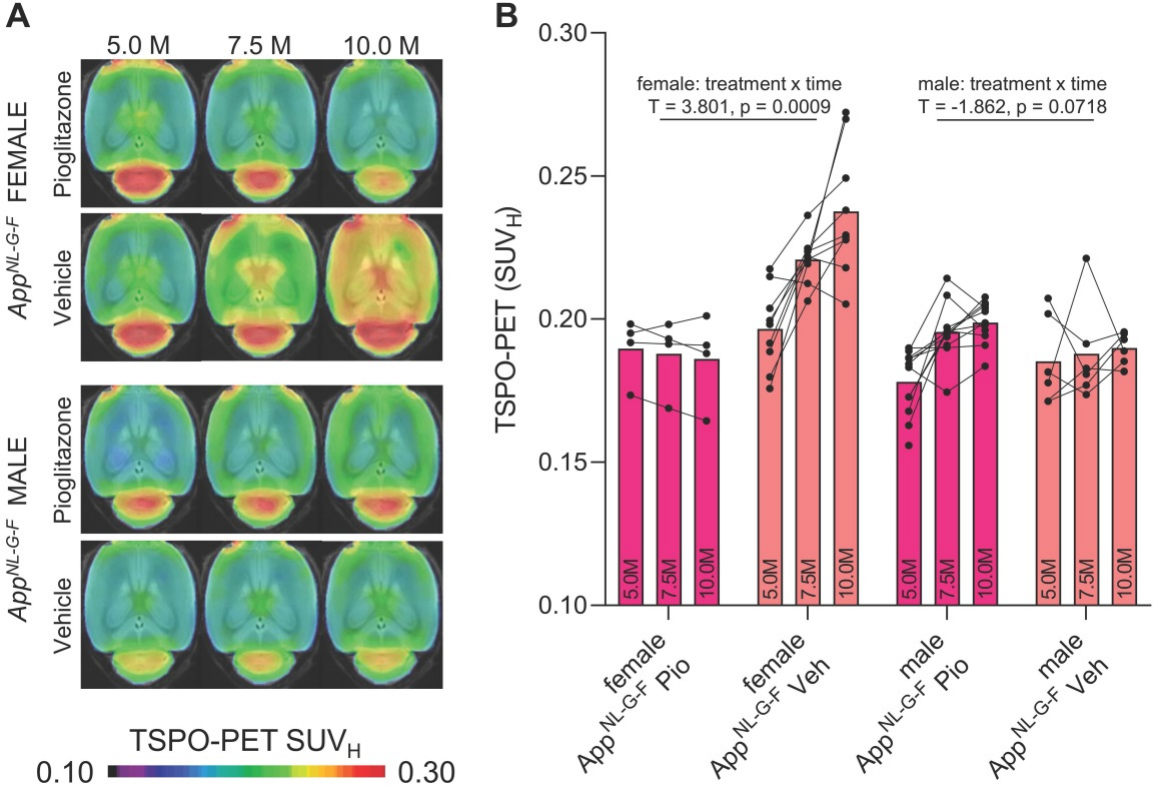
^18^F-GE-180 TSPO-PET monitoring of effects of chronic pioglitazone treatment in female and male *App^NL-G-F^* mice. (**A**) Axial images show group means of the TSPO-PET signal (myocardium scaled standardized uptake value, SUV_H_) at different ages in treatment and vehicle groups, projected upon a standard MRI template. Baseline scans were performed prior to therapy initiation. (**B**) Individual time courses of the cortical TSPO-PET signal during the pharmacological treatment period. Statistics derive from a linear mixed model. Female *App^NL-G-F^* pioglitazone n = 4, female *App^NL-G-F^* vehicle n = 9, male *App^NL-G-F^* pioglitazone n = 11, male *App^NL-G-F^* vehicle n = 6. Pio = pioglitazone treatment, Veh = vehicle treatment.

Next, we tested whether previously observed sex differences in TSPO expression in mouse brain 29 have an impact on the responses to PPARγ pharmacological stimulation. To this end, we used the novel APP knock-in model* App^NL-G-F^* 16 mice and performed longitudinal TSPO-PET imaging during chronic pioglitazone treatment in groups of female and male mice. Furthermore, we tested whether these mice showed effects of PPARγ stimulation on the TSPO-PET signal in the absence of APP overexpression. Baseline levels of the TSPO-PET signal in female *App^NL-G-F^* mice at 5 months of age were lower compared to baseline levels of female PS2APP mice at 8 months of age (-21%, p < 0.0001; Figure S1). We observed sex-specific elevation of the TSPO-PET signal in vehicle-treated female *App^NL-G-F^* mice when compared to males aged 7.5 (+18%, p = 0.017) and 10 months (+25%, p = 0.0007; sex × time interaction: b/SE = -0.100/0.030, T = -3.273, p = 0.0003, linear mixed model controlling for random intercept). Female *App^NL-G-F^* mice with pioglitazone treatment showed a smaller TSPO-PET signal increase compared to vehicle-treated female *App^NL-G-F^* mice aged from five to ten months (**Figure 2**). This resulted from an attenuated TSPO-PET signal increase in pioglitazone-treated female *App^NL-G-F^* mice aged 7.5 (-15%, p = 0.030) and 10 months (-21%, p = 0.0053) when compared to vehicle-treated female *App^NL-G-F^* mice (treatment × time interaction: b/SE = 0.114/0.030, T = 3.801, p = 0.0009, linear mixed model controlling for random intercept, **Figure 2**). On the other hand, male *App^NL-G-F^* mice with pioglitazone treatment tended to show a slight exaggerated increase of the TSPO-PET signal from five to ten months of age when compared to vehicle treated male *App^NL-G-F^* mice (+12% vs. +2%, treatment × time interaction: b/SE = -0.041/0.022, T = -1.862, p = 0.072). Wild-type mice did not show differences in TSPO-PET signal between treated or non-treated animals for this time span. Baseline levels of Aβ-PET and the Aβ-PET rate of change did not differ between female and male *App^NL-G-F^* mice (Figure S1).

### Baseline TSPO-PET predicts treatment associated changes in microglial activation during chronic PPARγ stimulation

**Figure 3:**
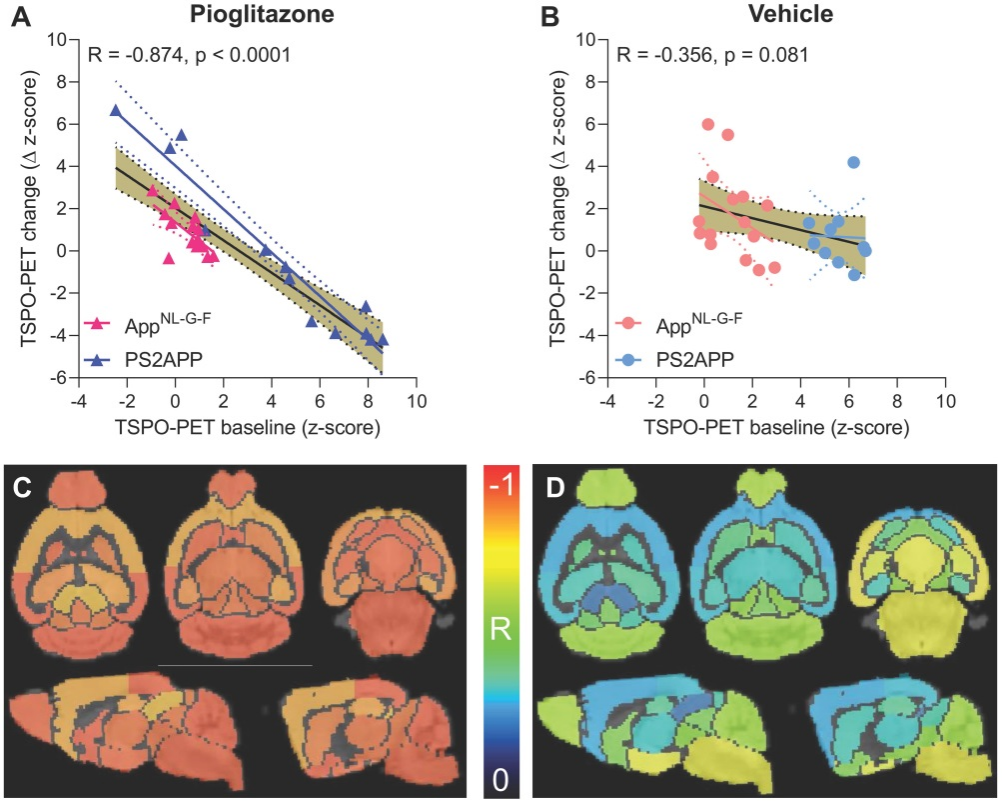
Prediction of changes in microglial activity by the ^18^F-GE-180 TSPO-PET baseline examination. (**A, B**) Correlation analysis between the TSPO-PET z-score at baseline and the change of the TSPO-PET z-score (baseline to last follow-up) in *App^NL-G-F^* and PS2APP mice with pioglitazone treatment (**A**) and vehicle controls (**B**). Correlations are illustrated for the combination of both mouse models (gold) and in separate analyses of *App^NL-G-F^* (red) and PS2APP (blue) mice. (**C, D**) Multiregional analysis of the correlation between the TSPO-PET z-score at baseline and the change of the TSPO-PET z-score (baseline to last follow-up). Re-projected coefficients of correlation (R) are illustrated in axial and sagittal slices projected upon a standard MRI template. *App^NL-G-F^* and PS2APP mice were analyzed together for the pioglitazone treatment group (**C**) and vehicle controls (**D**). Levels of significance per region after false discovery rate correction for multiple comparisons are provided in **Table 1**.

Given the observed heterogeneity of changes in TSPO-PET after induction of PPARγ pharmacological stimulation, we asked if TSPO-PET at baseline serves to predict the individual longitudinal changes in microglial activation upon treatment. Strikingly, we observed a strong negative association between baseline TSPO-PET and subsequent changes in the TSPO-PET signal across pioglitazone treated animals (R = -0.874, p < 0.001, **Figure 3A-B**), suggesting that mice with high microglial activation at baseline respond more strongly to PPARγ stimulation. Importantly, this association was also present in independent cohorts of PS2APP mice (R = -0.964, p < 0.0001) and *App^NL-G-F^* mice (R = -0.680, p = 0.0053) mice with chronic pioglitazone treatment. On the other hand, there was only a trend towards a negative association between the baseline TSPO-PET signal and subsequent TSPO-PET changes in vehicle treated animals (R = -0.356, p = 0.081). The association between pre-therapeutic TSPO-PET results and changes in microglial activation of pioglitazone-treated mice was observed across all brain regions, with the strongest differences relative to vehicle treated mice in neocortical areas, hippocampus, striatum, and thalamus (**Figure 3C-D; Table 1**). Several subcortical regions also showed a significant negative association between the baseline TSPO-PET signal and changes in microglial activation in the vehicle cohort (**Table 1**), congruent with the observation that microglial activation at baseline *per se* has a predictive value for longitudinal alterations of microglial activity and spatial learning performance 20, 30.

### PPARγ stimulation induced changes of microglial activation predict spatial learning performance and aggregation of fibrillar Aβ

**Figure 4:**
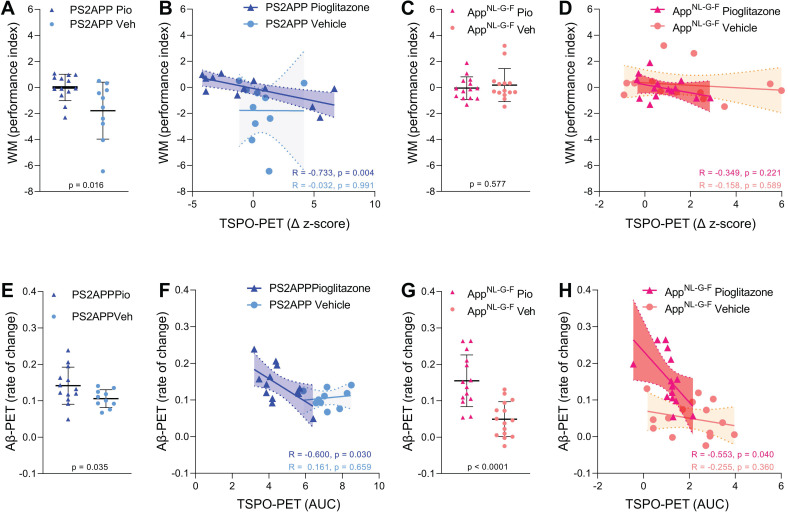
Associations of ^18^F-GE-180 TSPO-PET findings with spatial learning performance and Aβ accumulation. (**A**) Water maze (WM) performance index (principal component analysis, PCA, higher score means better performance) in the comparison of PS2APP mice after five months pioglitazone treatment and their vehicle controls. (**B**) Correlation between the longitudinal change of TSPO-PET in PS2APP mice and the water maze performance index. (**C**) Water maze performance index in the comparison of *App^NL-G-F^* mice after five months pioglitazone treatment and their vehicle controls. (**D**) Correlation between the longitudinal change of TSPO-PET in* App^NL-G-F^* mice and the water maze performance index. (**E**) Aβ-PET rate of change (Δ SUVR) in the comparison of PS2APP mice after five months pioglitazone treatment and their vehicle controls 12. (**F**) Correlation between the TSPO-PET rate of change in PS2APP mice and the Aβ-PET rate of change. (**G**) Aβ-PET rate of change (Δ SUVR) in the comparison of *App^NL-G-F^* mice after five months pioglitazone treatment and their vehicle controls 12. (**H**) Correlation between the TSPO-PET rate of change in* App^NL-G-F^* mice and the Aβ-PET rate of change. P-values of the group comparisons derive from an unpaired two-tailed t-test. R- and P-values of the correlation analyses derive from a Pearson correlation. PS2APP pioglitazone n = 13, PS2APP vehicle n = 10, *App^NL-G-F^* pioglitazone n = 15, *App^NL-G-F^* vehicle n = 15. AUC = area under the curve.

Next, we asked if altered TSPO expression during chronic pioglitazone treatment has associations with known determinants of therapeutic effects in the AD models. To this end, we correlated the rate of change in the TSPO-PET signal during the treatment period with the individual spatial learning impairment and changes in fibrillary Aβ pathology measured post mortem. Better spatial learning was associated with an attenuated increase of the TSPO-PET signal during five months of PPARγ stimulation in PS2APP mice (R = -0.733, p = 0.0043, **Figure 4A-B**), but the association did not reach statistical significance in *App^NL-G-F^* mice (R = -0.349, p = 0.221, **Figure 4C-D**). The observed effect in PS2APP mice was treatment-specific, since there was no association between altered TSPO expression and spatial learning in vehicle treated mice (R = -0.032, p = 0.991,** Figure 4B**). Our dedicated analysis of Aβ species during chronic PPARγ stimulation in this same cohort 12 revealed a greater increase in fibrillar Aβ, which is the major source of the Aβ-PET signal 31, in both treated mouse models compared to their vehicle controls, which reflected a shift of Aβ plaques towards a more fibrillary composition. Meanwhile, the non-fibrillar proportion of plaques decreased upon the treatment, as is reported elsewhere 12. A low area under the curve (AUC) of TSPO-PET signal during the recording period was associated with a higher rate of change of fibrillar Aβ in pioglitazone-treated PS2APP (R = -0.600, p = 0.030, **Figure 4E-F**) and* App^NL-G-F^* mice (R = -0.553, p = 0.040, **Figure 4G-H**). Vehicle controls of both models did not show significant associations between the TSPO-PET AUC and changes in fibrillary Aβ pathology.

### ^18^F-GE-180 TSPO-PET signal reflects activated microglia

Finally, we set about to elucidate the molecular source of the TSPO-PET signal. Earlier studies have already validated* in vivo* TSPO-PET as a microglial marker relative to immunohistochemistry *ex vivo* 10, 11 and we have elsewhere demonstrated that PPARγ-related modulation of microglia can be detected by terminal immunohistochemistry in these mouse models 12. However, the molecular and cellular correlates of altered TSPO expression during pharmacological PPARγ stimulation remained unclear. To establish this relationship, we performed an immunohistochemical validation of TSPO-PET in subpopulations of all study groups using antibodies against a general marker of microglia (Iba-1) and a specific marker of microglial activation (CD68).

**Figure 5:**
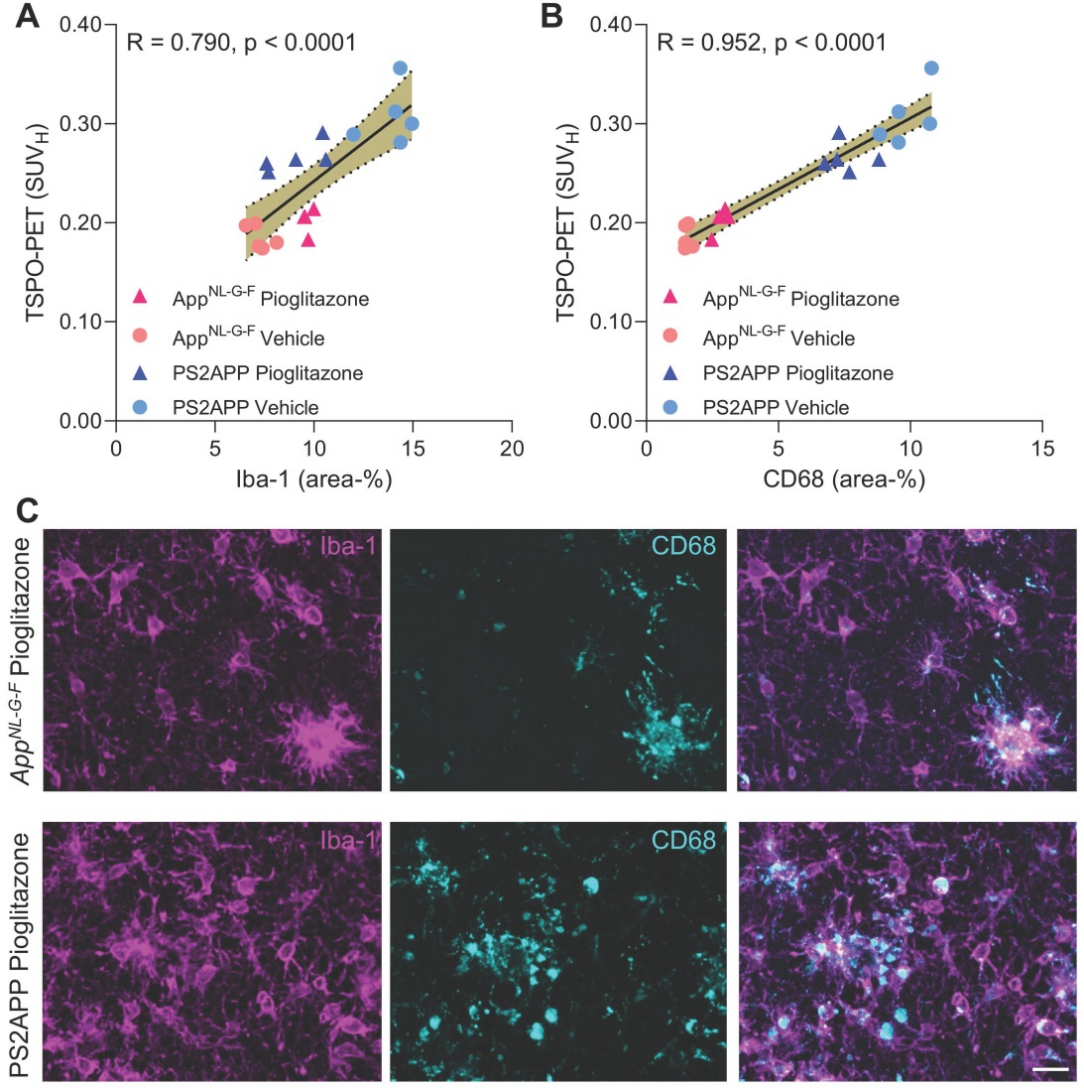
** A, B:** Correlation analysis between immunohistochemistry markers of microglial activation and ^18^F-GE-180 TSPO-PET at the terminal time-point. *App^NL-G-F^* pioglitazone n = 4, *App^NL-G-F^* n = 5, PS2APP pioglitazone n = 5, PS2APP vehicle n = 5. Error bands represent the 95% confidence intervals. SUV_H_ = standardized uptake value including myocardium correction. R = Pearson's coefficient of correlation. **C:** Representative immunohistochemistry images of treated *App^NL-G-F^* and PS2APP mice, indicating a similar Iba-1 area coverage but a higher CD68 area coverage of PS2APP mice compared to* App^NL-G-F^*. The differentiation by CD68 fitted to corresponding TSPO-PET images, clearly showing an elevated TSPO-PET signal in PS2APP mice compared to* App^NL-G-F^*. Scale bar = 20 µm.

Iba-1 (R = 0.790, p < 0.0001, **Figure 5A**) and CD68 (R = 0.952, p < 0.0001, **Figure 5B**) immunohistochemistry results correlated highly with TSPO-PET binding *in vivo*. Importantly, we saw a stronger association between TSPO-PET with CD68 labelling, which we attribute to the lesser differentiation of Iba-1 immunohistochemistry for treated *App^NL-G-F^* and PS2APP. Indeed, Iba-1 immunohistochemistry did not differentiate between treated *App^NL-G-F^* and treated PS2APP mice. The lacking differentiation of pioglitazone-treated PS2APP and *App^NL-G-F^* mice by Iba-1 immunohistochemistry was also discernible at the individual mouse level (**Figure 5C**)**.**

## Discussion

In this longitudinal study, we investigated serial TSPO-PET imaging as a tool for monitoring of chronic immunomodulation in two distinct mouse models of amyloidosis. Here, PET with the TSPO ligand ^18^F-GE-180 sensitively detected changes of microglial activity upon pharmacological PPARγ stimulation. Furthermore, we discovered an important sex difference in this treatment response. Pre-therapeutic TSPO-PET measures supported the prediction of individual treatment responses across mouse models and sexes, thus indicating that baseline TSPO expression has an association with the effect of immunomodulation. Immunohistochemistry results confirmed that TSPO-PET is sensitive to activated microglia in the present models.

Our results prove that PET with the TSPO ligand ^18^F-GE-180 can sensitively monitor pioglitazone-induced changes of microglial activity during chronic treatment of AD-model mice. This finding is important given that an earlier PET study using the less avid TSPO ligand ^11^C-(*R*)-PK11195 failed to detect treatment-induced changes in the TSPO-PET signal during chronic pioglitazone administration in APPPS1 mice 32. Nonetheless, the ^11^C-(*R*)-PK11195 methodology was sufficiently sensitive to detect microglial activation in transgenic versus wild-type mice. We note that a head-to-head comparison with an equal treatment setting would be required to draw robust conclusions on the superiority of one tracer over the other. However, earlier studies support our present findings of excellent sensitivity for ^18^F-GE-180 TSPO-PET, since the tracer outperformed ^11^C-(R)-PK11195 in a preclinical head-to-head comparison after lipopolysaccharide challenge 33 and revealed higher specific binding *in vivo* when compared to ^11^C-PBR23 in a human blocking study 34, 35. Importantly, we successfully measured treatment effects on microglial activation by TSPO-PET in two distinct Aβ mouse models. Here, our use of *App^NL-G-F^* mice 16 provided evidence that TSPO expression is altered by pharmacological PPARγ stimulation in mice without overexpression of APP. In line with our data, ^18^F-GE-180 PET also enabled the detection of *reduced* microglial activation during neurotrophin receptor modulation by LM11A-31 9. Furthermore,^ 18^F-GE-180 PET sensitively detected different temporal patterns of microglial activation upon administration of several anti-pyroglutamate-3 Aβ immunotherapy agents 36. In summary, our PET monitoring of pharmacological PPARγ stimulation clarified that the direct modulation of microglial activity can be captured *in vivo*.

The main finding of our study is that pre-therapeutic and serial TSPO-PET recordings in our chronic pioglitazone treatment paradigm were closely associated with the treatment response. First, mice with high microglial activation at baseline showed stronger treatment effects, which was underpinned by a strong association between high baseline TSPO-PET quantitation and slower rate of increase in the TSPO-PET signal during the five months of PPARγ stimulation. Thus, TSPO-PET imaging of microglial activation may potentially serve as a translational tool 1 that could allow for predictive response stratification before or during immunomodulation in the context of precision medicine. However, we note that lacking standardization of radiotracers and their quantification are hurdles that must be overcome to enable successful translation to human studies. Second, the magnitude of microglial activation during the treatment period had close associations with changes in fibrillar Aβ pathology of both models and with spatial learning performance of PS2APP mice. The present finding of stronger increases of fibrillar Aβ in mice with low baseline microglial activity is entirely in line with our translational study in mice with amyloidosis and AD patients 28. Thus, the present results strengthen the hypothesis that it is activated microglia that mediate the clearance of excess fibrillar Aβ. Interpretation of the observed association between low microglial activation and a behavioural source of better spatial learning performance calls for some caution. Although a sufficient microglial response seems important to maintain brain function in therapy-naive AD model mice 20, the suppression of microglial activation by PPARγ stimulation was directly correlated with better spatial learning performance in the current study. Thus, we suppose that PPARγ stimulation shifted the already activated microglia (i.e., in mice with high TSPO-PET levels at baseline) towards a more pronounced neuroprotective function. Proving this conjecture might call for a more rigorous discrimination of the M1/M2 phenotypic characteristics than is afforded by present TSPO-PET radiotracers. Nonetheless, present data definitely substantiate that microglia play a major role in the histological and behavioral consequences of cerebral amyloidosis in mice.

Interestingly, we observed a pronounced sex effect on the pioglitazone treatment response in *App^NL-G-F^* mice. Vehicle-treated female *App^NL-G-F^* mice showed the previously reported stronger increase of TSPO expression when compared to their male littermates 29, but PPARγ stimulation attenuated this increase in females while tending to exacerbate the course of neuroinflammation in males. This finding may be of remarkable significance, since some pioglitazone studies used only female mice 37 or did not declare the sex of mice 38. Thus, potential sex effects of PPARγ stimulation might have been missed in these studies. Furthermore, this sex effect merits attention consideration in planning human studies, since levels of sex hormones can impact upon microglial modulation 39. On the other hand, our parallel detailed analysis of amyloid aggregation during chronic PPARγ stimulation in this cohort did not show relevant sex differences in the rate of increase in Aβ PET signal 12. Still, the present results fit with our previously reported dependency of the Aβ-PET rate of change in microglial activation in AD model mice 28, since pioglitazone treatment in male and female *App^NL-G-F^* mice resulted in similar microglia activation levels at the end of the study. Interestingly, a study using the same TSPO radiotracer similarly found a specific response of the PET signal in female APP/PS1dE9 mice to ^56^Fe radiation 40. Thus, sex differences need to be taken into consideration when planning TSPO-PET imaging studies of Aβ mouse models. The small number of female mice treated with pioglitazone and the unbalanced comparison of female and male *App^NL-G-F^* mice must be considered as limitations of the current study. Thus, the present results should stimulate confirmatory studies in different Aβ and tau mouse models together with studies involving manipulation of sex hormones, aiming to describe more mechanistically the observed sex differences.

We initiated PPARγ therapy at ages manifesting an early phase of limited fibrillar amyloidosis in both mouse models, thus emulating an early but detectable stage of the human AD continuum 41. In consideration of emerging plasma biomarkers for AD pathology 42, novel treatments for AD shall likely be initiated at a comparable disease stage in future clinical studies. Thus, we focused our intervention monitoring on the phase of amyloid aggregation, which revealed the greatest therapeutic response in those AD model mice with a seemingly more aggressive microglial activation during the early amyloid build-up phase. Thus, insofar as PET, cerebrospinal fluid, or plasma biomarkers of microglial activation could serve for treatment stratification in patients with early AD that have a positive Aβ-status, we foresee opportunities emerging for personalized precision medicine. The major drawback of current mouse AD models is the missing conversion of a sole Aβ-positive stage (A+T-) to combined Aβ/tau-positivity (A+T+). Although the recent literature describes novel combinations of Aβ/tau gene modification 43, 44, these models still do not present a breakthrough in better mimicking human AD. Conceivably, cortical tau seeding in an Aβ mouse model might yield a more AD-like model of tauopathy, but such models are not yet ready for large scaled testing of drugs 45. Thus, we note as a limitation of the present study that we were unable to investigate effects of pioglitazone on conversion to tau-positivity or during subsequent tau spreading. As a consequence, we cannot predict the efficacy of chronic PPARγ stimulation on the tauopathy encountered at late stages of AD.

The molecular sources of the TSPO-PET signal in neurodegenerative diseases remained to be fully elucidated 35. We undertook a correlation analysis between TSPO-PET and immunohistochemistry endpoints in heterogeneous samples of two mouse models, factoring for age, sex, and presence or absence of immunomodulation. Here, we found that the activated microglial marker CD68 proved to have a much better correlation with TSPO-PET signal. In contrast, Iba-1 immunohistochemistry did not distinguish between PS2APP and *App^NL-G-F^* mice after pioglitazone treatment, although the two groups were clearly separated by TSPO-PET and CD68 immunohistochemistry. This finding is in line with the first ^18^F-GE-180 study in rodent models of AD which showed a co-localization between TSPO and CD68 46. Thus, TSPO-PET is very sensitive to detect disease-associated microglial activation, which also fits to the strong correlations between CD68 and TSPO-PET reported in Trem2-deficient APPPS1 mice 11. As a major limitation, we note that we cannot draw detailed conclusions on the specificity of the TSPO-PET signal since we did not measure associations with reactive astrocytosis to GFAP immunohistochemistry. Furthermore, although we standardized isoflurane levels across all genotype and treatment groups, we cannot exclude a general impact of isoflurane on the variance in TSPO-PET 47. Effects of anesthesia on TSPO-PET in mice may hamper the translation to human data since patients are only very rarely imaged under general anesthesia.

PPARγ receptor agonists represent a rather unspecific drug since PPARγ is involved in various pathways in addition to peroxisome activation, notably including glucose metabolism and insulin sensitization 48. We selected pioglitazone for immunomodulation of microglial activity in AD mouse models as the effects of this drug are well understood. Nonetheless, more specific drugs like NLRP3 regulators 49 could enable a more direct targeting of the inflammasome in neurodegenerative diseases. Optimization of immunomodulation strategies could potentially improve their effectiveness and reduce their side effects, whereupon our present TSPO-PET imaging paradigm would be readily transferable to other drugs, so long as they target activated microglia. Ultimately, specific radioligands for different microglia phenotypes 50 could enhance the monitoring of immunomodulation *in vivo*. However, it needs to be considered that the transcriptome of human microglia is more complex than that of rodent microglia 51. This implies that development and translation of radiotracers for specific microglia phenotypes need to be conducted carefully and in close collaboration with transcriptome sequencing experts.

## Conclusion

TSPO-PET serves as a sensitive biomarker for *in vivo* monitoring of immunomodulation in mouse AD models. Pre-therapeutic assessment of microglial activation in individual mice was associated with the response to immunomodulation therapy, indicating that a biomarker of microglial activation could predict treatment effects. There were pronounced sex differences in the responses to PPARγ stimulation effects *in vivo*. The observed heterogeneity of treatment responses in mice with equal genetic background calls for testing of similar concepts in the design of biomarker studies assessing effects of immunomodulation on microglial activation in translational trials in AD patients.

## Supplementary Material

Supplemental Figure S1.

## Figures and Tables

**Table 1:** Multi-region analysis of baseline prediction of longitudinal microglial activation by baseline TSPO-PET

Region	Pioglitazone		Vehicle		Contrast
	R	p-value (FDR-corrected)	R	p-value (FDR-corrected)	ΔR
Striatum R	-0.941	4.9E-13***	-0.427	0.12	0.514
Striatum L	-0.899	1.0E-10***	-0.342	0.12	0.557
Hippocampus R	-0.879	8.6E-10***	-0.344	0.12	0.535
Hippocampus L	-0.854	8.6E-09***	-0.388	0.083	0.466
Thalamus	-0.920	7.8E-12***	-0.326	0.13	0.594
Cerebellum	-0.952	1.4E-13***	-0.584	0.0066**	0.368
Basal forebrain & septum	-0.949	1.4E-13***	-0.516	0.017*	0.433
Hypothalamus	-0.936	8.6E-13***	-0.705	0.0009***	0.231
Amygdala R	-0.935	8.6E-13***	-0.665	0.0012**	0.270
Amygdala L	-0.935	9.0E-13***	-0.666	0.0015**	0.269
Brainstem	-0.943	1.4E-12***	-0.691	0.0009***	0.252
Central grey	-0.914	1.8E-11***	-0.505	0.019*	0.409
Superior colliculi	-0.804	2.6E-07***	-0.199	0.34	0.605
Olfactory bulb	-0.938	7.5E-13***	-0.603	0.0050**	0.335
Midbrain R	-0.919	9.5E-12***	-0.558	0.0099**	0.361
Midbrain L	-0.922	6.4E-12***	-0.541	0.012*	0.381
Inferior colliculus R	-0.900	3.0E-11***	-0.486	0.11	0.414
Inferior colliculus L	-0.910	9.7E-11***	-0.357	0.024*	0.553
Piriform/entorhinal cortex	-0.883	6.2E-10***	-0.742	0.0005***	0.141
Auditory/visual cortex	-0.947	1.8E-13***	-0.306	0.15	0.641
Motor/sensory cortex	-0.836	3.2E-08***	-0.283	0.18	0.553

Person's correlation coefficients (R) were calculated between baseline TSPO-PET (z-score) and the change in TSPO-PET (Δ z-score) during the five months treatment period in pioglitazone and vehicle treated *App^NL-G-F^* and PS2APP mice. P-values were adjusted for multiple comparisons by false discovery rate correction. *p < 0.05; **p < 0.01; ***p < 0.001.

## References

[1] Heneka MT, Carson MJ, Khoury JE, Landreth GE, Brosseron F, Feinstein DL (2015). Neuroinflammation in Alzheimer's disease. Lancet Neurol.

[2] Fakhoury M (2018). Microglia and astrocytes in Alzheimer's disease: Implications for therapy. Curr Neuropharmacol.

[3] Ahmad MH, Fatima M, Mondal AC (2019). Influence of microglia and astrocyte activation in the neuroinflammatory pathogenesis of Alzheimer's disease: Rational insights for the therapeutic approaches. J Clin Neurosci.

[4] Zou C, Shi Y, Ohli J, Schuller U, Dorostkar MM, Herms J (2016). Neuroinflammation impairs adaptive structural plasticity of dendritic spines in a preclinical model of Alzheimer's disease. Acta Neuropathol.

[5] Mandrekar-Colucci S, Karlo JC, Landreth GE (2012). Mechanisms underlying the rapid peroxisome proliferator-activated receptor-gamma-mediated amyloid clearance and reversal of cognitive deficits in a murine model of Alzheimer's disease. J Neurosci.

[6] Burns DK, Chiang C, Welsh-Bohmer KA, Brannan SK, Culp M, O'Neil J (2019). The TOMMORROW study: Design of an Alzheimer's disease delay-of-onset clinical trial. Alzheimers Dement (N Y).

[7] Scott G, Zetterberg H, Jolly A, Cole JH, De Simoni S, Jenkins PO (2018). Minocycline reduces chronic microglial activation after brain trauma but increases neurodegeneration. Brain.

[8] Wolf BJ, Brackhan M, Bascunana P, Leiter I, Langer BLN, Ross TL (2020). TSPO PET identifies different anti-inflammatory minocycline treatment response in two rodent models of epileptogenesis. Neurotherapeutics.

[9] James ML, Belichenko NP, Shuhendler AJ, Hoehne A, Andrews LE, Condon C (2017). [(18)F]GE-180 PET detects reduced microglia activation after LM11A-31 therapy in a mouse model of Alzheimer's disease. Theranostics.

[10] Brendel M, Probst F, Jaworska A, Overhoff F, Korzhova V, Albert NL (2016). Glial activation and glucose metabolism in a transgenic amyloid mouse model: A triple-tracer PET study. J Nucl Med.

[11] Parhizkar S, Arzberger T, Brendel M, Kleinberger G, Deussing M, Focke C (2019). Loss of TREM2 function increases amyloid seeding but reduces plaque-associated ApoE. Nat Neurosci.

[12] Blume T, Deussing M, Biechele G, Peters F, Zott B, Schmidt C (2021). Chronic PPARγ stimulation shifts amyloidosis to higher fibrillarity but improves cognition. bioRxiv.

[13] Richards JG, Higgins GA, Ouagazzal AM, Ozmen L, Kew JN, Bohrmann B (2003). PS2APP transgenic mice, coexpressing hPS2mut and hAPPswe, show age-related cognitive deficits associated with discrete brain amyloid deposition and inflammation. J Neurosci.

[14] Ozmen L, Albientz A, Czech C, Jacobsen H (2009). Expression of transgenic APP mRNA is the key determinant for beta-amyloid deposition in PS2APP transgenic mice. Neurodegener Dis.

[15] Masuda A, Kobayashi Y, Kogo N, Saito T, Saido TC, Itohara S (2016). Cognitive deficits in single App knock-in mouse models. Neurobiol Learn Mem.

[16] Saito T, Matsuba Y, Mihira N, Takano J, Nilsson P, Itohara S (2014). Single App knock-in mouse models of Alzheimer's disease. Nat Neurosci.

[17] Sacher C, Blume T, Beyer L, Peters F, Eckenweber F, Sgobio C (2019). Longitudinal PET monitoring of amyloidosis and microglial activation in a second-generation amyloid-beta mouse model. J Nucl Med.

[18] Overhoff F, Brendel M, Jaworska A, Korzhova V, Delker A, Probst F (2016). Automated spatial brain normalization and hindbrain white matter reference tissue give improved [(18)F]-florbetaben PET quantitation in Alzheimer's model mice. Front Neurosci.

[19] Deussing M, Blume T, Vomacka L, Mahler C, Focke C, Todica A (2017). Coupling between physiological TSPO expression in brain and myocardium allows stabilization of late-phase cerebral [(18)F]GE180 PET quantification. Neuroimage.

[20] Focke C, Blume T, Zott B, Shi Y, Deussing M, Peters F (2019). Early and longitudinal microglial activation but not amyloid accumulation predicts cognitive outcome in PS2APP mice. J Nucl Med.

[21] Schiffer WK, Mirrione MM, Biegon A, Alexoff DL, Patel V, Dewey SL (2006). Serial microPET measures of the metabolic reaction to a microdialysis probe implant. J Neurosci Methods.

[22] Biechele G, Wind K, Blume T, Sacher C, Beyer L, Eckenweber F Microglial activation in the right amygdala-entorhinal-hippocampal complex is associated with preserved spatial learning in App(NL-G-F) mice. Neuroimage. 2020: 117707.

[23] Sauvage M, Brabet P, Holsboer F, Bockaert J, Steckler T (2000). Mild deficits in mice lacking pituitary adenylate cyclase-activating polypeptide receptor type 1 (PAC1) performing on memory tasks. Brain Res Mol Brain Res.

[24] Busche MA, Kekus M, Adelsberger H, Noda T, Forstl H, Nelken I (2015). Rescue of long-range circuit dysfunction in Alzheimer's disease models. Nat Neurosci.

[25] Keskin AD, Kekus M, Adelsberger H, Neumann U, Shimshek DR, Song B (2017). BACE inhibition-dependent repair of Alzheimer's pathophysiology. Proc Natl Acad Sci U S A.

[26] Bromley-Brits K, Deng Y, Song W (2011). Morris water maze test for learning and memory deficits in Alzheimer's disease model mice. J Vis Exp.

[27] Eckenweber F, Medina-Luque J, Blume T, Sacher C, Biechele G, Wind K (2020). Longitudinal TSPO expression in tau transgenic P301S mice predicts increased tau accumulation and deteriorated spatial learning. J Neuroinflammation.

[28] Ewers M, Biechele G, Suarez-Calvet M, Sacher C, Blume T, Morenas-Rodriguez E (2020). Higher CSF sTREM2 and microglia activation are associated with slower rates of beta-amyloid accumulation. EMBO Mol Med.

[29] Biechele G, Franzmeier N, Blume T, Ewers M, Luque JM, Eckenweber F (2020). Glial activation is moderated by sex in response to amyloidosis but not to tau pathology in mouse models of neurodegenerative diseases. J Neuroinflammation.

[30] Blume T, Focke C, Peters F, Deussing M, Albert NL, Lindner S (2018). Microglial response to increasing amyloid load saturates with aging: a longitudinal dual tracer *in vivo* muPET-study. J Neuroinflammation.

[31] Biechele G, Sebastian Monasor L, Wind K, Blume T, Parhizkar S, Arzberger T (2021). Glitter in the darkness? Non-fibrillar beta-amyloid plaque components significantly impact the beta-amyloid PET signal in mouse models of Alzheimer's disease. J Nucl Med.

[32] Rapic S, Backes H, Viel T, Kummer MP, Monfared P, Neumaier B (2013). Imaging microglial activation and glucose consumption in a mouse model of Alzheimer's disease. Neurobiol Aging.

[33] Dickens AM, Vainio S, Marjamaki P, Johansson J, Lehtiniemi P, Rokka J (2014). Detection of microglial activation in an acute model of neuroinflammation using PET and radiotracers 11C-(R)-PK11195 and 18F-GE-180. J Nucl Med.

[34] Sridharan S, Raffel J, Nandoskar A, Record C, Brooks DJ, Owen D (2019). Confirmation of specific binding of the 18-kDa translocator protein (TSPO) radioligand [(18)F]GE-180: a blocking study using XBD173 in multiple sclerosis normal appearing white and grey matter. Mol Imaging Biol.

[35] Cumming P, Burgher B, Patkar O, Breakspear M, Vasdev N, Thomas P (2018). Sifting through the surfeit of neuroinflammation tracers. J Cereb Blood Flow Metab.

[36] Crehan H, Liu B, Kleinschmidt M, Rahfeld JU, Le KX, Caldarone BJ (2020). Effector function of anti-pyroglutamate-3 Abeta antibodies affects cognitive benefit, glial activation and amyloid clearance in Alzheimer's-like mice. Alzheimers Res Ther.

[37] Dorostkar MM, Burgold S, Filser S, Barghorn S, Schmidt B, Anumala UR (2014). Immunotherapy alleviates amyloid-associated synaptic pathology in an Alzheimer's disease mouse model. Brain.

[38] Seok H, Lee M, Shin E, Yun MR, Lee YH, Moon JH (2019). Low-dose pioglitazone can ameliorate learning and memory impairment in a mouse model of dementia by increasing LRP1 expression in the hippocampus. Sci Rep.

[39] Pike CJ (2017). Sex and the development of Alzheimer's disease. J Neurosci Res.

[40] Liu B, Hinshaw RG, Le KX, Park MA, Wang S, Belanger AP (2019). Space-like (56)Fe irradiation manifests mild, early sex-specific behavioral and neuropathological changes in wildtype and Alzheimer's-like transgenic mice. Sci Rep.

[41] Jack CR Jr, Bennett DA, Blennow K, Carrillo MC, Dunn B, Haeberlein SB (2018). NIA-AA Research Framework: Toward a biological definition of Alzheimer's disease. Alzheimers Dement.

[42] Bateman RJ, Blennow K, Doody R, Hendrix S, Lovestone S, Salloway S (2019). Plasma biomarkers of AD emerging as essential tools for drug development: An EU/US CTAD task force report. J Prev Alzheimers Dis.

[43] Saito T, Mihira N, Matsuba Y, Sasaguri H, Hashimoto S, Narasimhan S (2019). Humanization of the entire murine Mapt gene provides a murine model of pathological human tau propagation. J Biol Chem.

[44] Cohen RM, Rezai-Zadeh K, Weitz TM, Rentsendorj A, Gate D, Spivak I (2013). A transgenic Alzheimer rat with plaques, tau pathology, behavioral impairment, oligomeric abeta, and frank neuronal loss. J Neurosci.

[45] He Z, Guo JL, McBride JD, Narasimhan S, Kim H, Changolkar L (2018). Amyloid-beta plaques enhance Alzheimer's brain tau-seeded pathologies by facilitating neuritic plaque tau aggregation. Nat Med.

[46] Liu B, Le KX, Park MA, Wang S, Belanger AP, Dubey S (2015). *In vivo* detection of age- and disease-related increases in neuroinflammation by 18F-GE180 TSPO microPET imaging in wild-type and Alzheimer's transgenic mice. J Neurosci.

[47] Saba W, Goutal S, Kuhnast B, Dolle F, Auvity S, Fontyn Y (2015). Differential influence of propofol and isoflurane anesthesia in a non-human primate on the brain kinetics and binding of [(18)F]DPA-714, a positron emission tomography imaging marker of glial activation. Eur J Neurosci.

[48] Tyagi S, Gupta P, Saini AS, Kaushal C, Sharma S (2011). The peroxisome proliferator-activated receptor: A family of nuclear receptors role in various diseases. J Adv Pharm Technol Res.

[49] Swanson KV, Deng M, Ting JP (2019). The NLRP3 inflammasome: molecular activation and regulation to therapeutics. Nat Rev Immunol.

[50] Götzl JK, Brendel M, Werner G, Parhizkar S, Sebastian Monasor L, Kleinberger G Opposite microglial activation stages upon loss of PGRN or TREM2 result in reduced cerebral glucose metabolism. EMBO Molecular Medicine. 2019: e9711.

[51] Gerrits E, Heng Y, Boddeke E, Eggen BJL (2020). Transcriptional profiling of microglia; current state of the art and future perspectives. Glia.

