# Supplementary material for: Pre-therapeutic microglia activation and sex determine therapy effects of chronic immunomodulation
Source: Theranostics. 2021 Aug 19;11(18):8964–76. doi: 10.7150/thno.64022 (PMC8419052; doi:10.7150/thno.64022)
Supplement: Supplementary file 1 — Supplemental Figure S1. [file thnov11p8964s1.pdf]

Supplement

Supplemental Figure 1

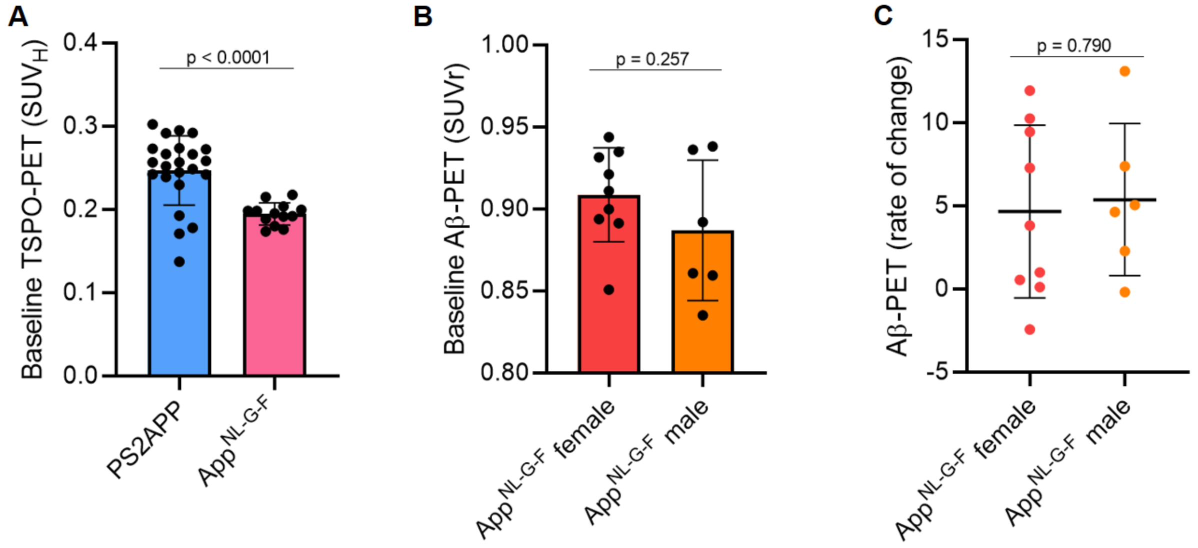

Figure S1 - Specific comparisons of TSPO-PET and Aβ-PET signals: (**A**) Comparison of baseline levels of microglial activation between PS2APP and to *App*$^{NL-G-F}$ mice. (**B, C**) Comparison of baseline Aβ-PET qunatification and the Aβ-PET rate of change between therapy naïve female and male *App*$^{NL-G-F}$ mice. P-values derive from an unpaired Students *t*-test.
